# Spontaneous resolution of tuberculosis related Rasmussen aneurysm in a young West African: A case report

**DOI:** 10.1016/j.radcr.2023.06.049

**Published:** 2023-08-05

**Authors:** Kwasi Adjepong Twum, Yaa Achiaa Afreh, Adu Tutu Amankwa, Kwasi Ankomah, Caroline Oku, Esi de Graft-Johnson

**Affiliations:** a Komfo Anokye Teaching Hospital (KATH). P.O. Box 1934, Kumasi, Ghana; b Department of Radiology, School of Medicine and Dentistry, Kwame Nkrumah University of Science and Technology (KNUST), Private Mail Bag, University Post Office, Kumasi, Ghana

**Keywords:** Tuberculosis, Rasmussen aneurysm, Hemoptysis, Case report

## Abstract

Rasmussen aneurysm (RA) is an uncommon cause of hemoptysis in pulmonary tuberculosis, first described in 1868 by Rasmussen. It is often treated with surgery or endovascular coiling. A few cases of spontaneous resolution with conservative management have been recorded in literature.

We present the case of a 44-year old patient who reported with hemoptysis, weight loss, chronic cough and night sweats and was diagnosed of pulmonary tuberculosis on the basis of clinical assessment and chest X-ray. Subsequently, chest CT scan done showed a giant left RA, treated conservatively with antituberculous chemotherapy with complete radiological resolution of aneurysm after 18-month follow-up.

We conclude that conservative management of RA is a good alternative in a low resource setting for hemodynamically stable patients.

## Introduction

Tuberculosis (TB) as a communicable disease is one of the world's leading causes of death and a significant contributor to poor health. Prior to the coronavirus (COVID-19) pandemic, TB rather than HIV/AIDS was the most common cause of infectious death globally [1]. Case detection rate for tuberculosis in Ghana in 2020 was 29% [1].

There are several manifestations and complications of pulmonary TB, including consolidation, lymph node enlargement, nodules (granulomas) and cavities, pleural effusion and lung collapse, acute respiratory distress syndrome, multiple cystic lesions resembling bullae and pneumatoceles, empyema necessitans, lung fibrosis and Rasmussen aneurysm (RA) [2,3].

RA are pulmonary artery branch pseudo-aneurysms caused by an adjacent infectious/inflammatory process weakening the adjacent arterial wall [4,5]. Most hemoptysis cases are caused by hypertrophied bronchial arteries whereas a few occurrences of hemoptysis result from pulmonary artery aneurysms [6].

We present a case of a young adult West African patient who presented with hemoptysis secondary to RA diagnosed on chest CT scan with spontaneous resolution after anti-TB chemotherapy.

## Case presentation

**Fig. 1:**
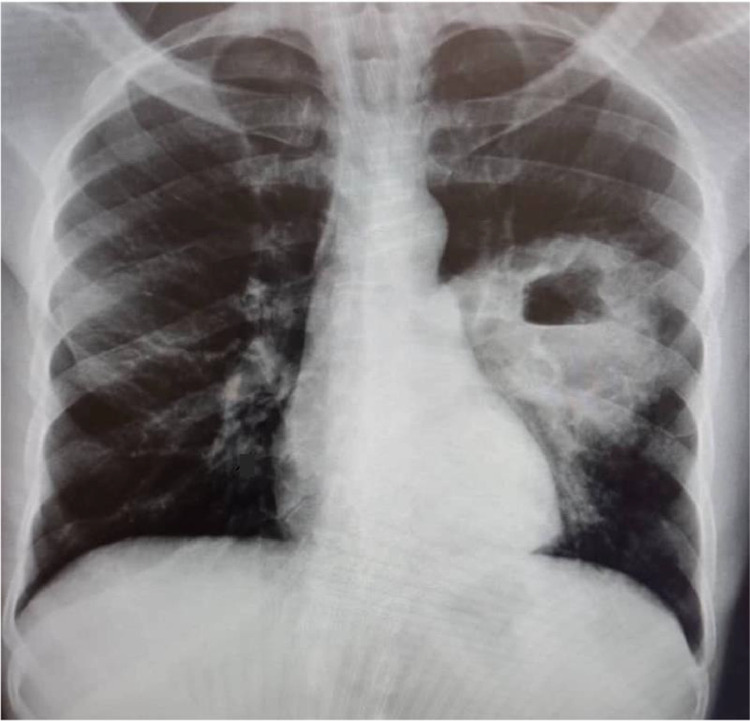
PA Erect Chest X-ray shows left middle zone thick-walled cavity with an internal air-fluid level and surrounding consolidation.

A 44-year-old patient from West Africa presented with a 1-month history of cough, fever, weight loss, and night sweats, along with a week of hemoptysis. The examination revealed a wasted young adult with digital clubbing. Chest auscultation showed bilateral bronchial breath sounds. A chest X-ray and sputum test for GeneXpert (a cartridge based nucleic acid amplification test for tuberculosis) were ordered. The sputum tests for acid-fast bacilli and GeneXpert both came back negative for TB. However, the erect chest X-ray revealed consolidation in the left middle zone and a thick-walled cavity with an air-fluid level (Fig. 1). The rest of the lung fields appeared normal with no pleural effusions. The patient was presumed to have pulmonary TB based on the chest X-ray and clinical findings. They were started on anti-TB chemotherapy (Isoniazid, Rifampicin, Pyrazinamide, and Ethambutol) and referred for a chest CT scan a week later.

**Fig. 2:**
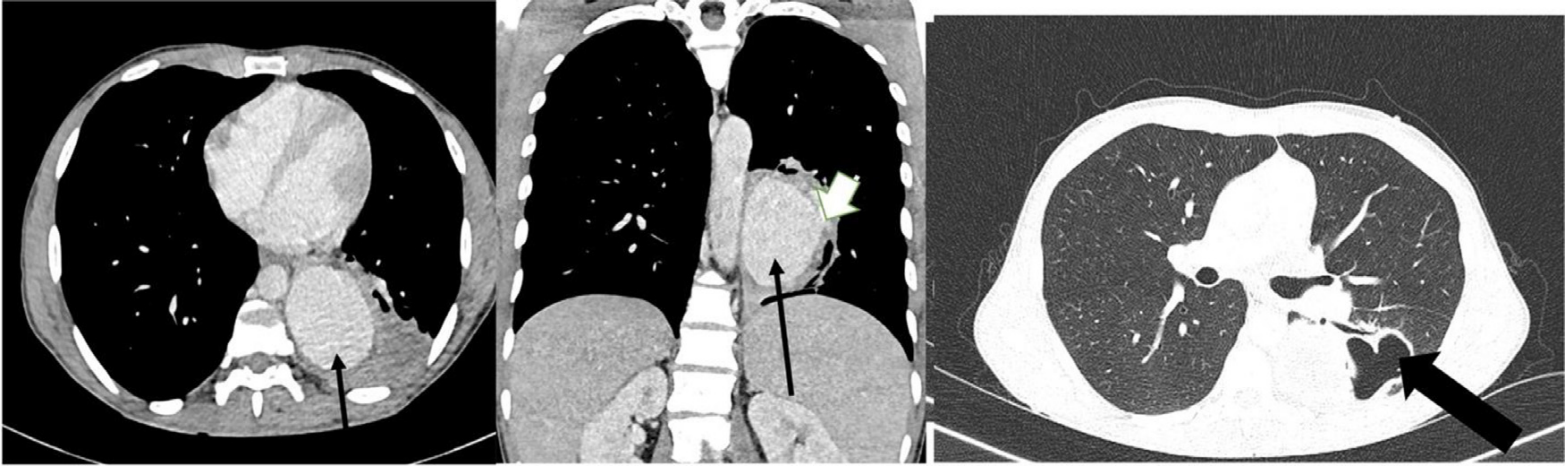
One-week post-treatment chest CT scan. Contrast enhanced chest CT scan shows a saccular aneurysmal dilation (straight arrow in A and B) of the left lower lobe segmental pulmonary artery (short broad arrow in B) measuring 7.0 (TRV) x 5.8 (AP) x 7.0 (CC) cm with an adjacent irregular cavitary lesion (long broad arrow in C).

The patient was a trader with no significant past medical or surgical history. There was no known contact with anyone diagnosed with TB or anyone with a chronic cough. The fever and night sweats resolved within 1 week of starting treatment. The chest CT scan, performed a week after the initial presentation, shows a saccular aneurysmal dilatation of the left lower lobe segmental artery measuring 7.0 (transverse [TRV]) x 5.8 (anteroposterior [AP]) x 7.0 (craniocaudal [CC]) cm with an adjacent irregular cavitary lesion. Surrounding consolidation was also seen in the superior and posterior segments of the left lower lobe, along with a hypodense nonenhancing area suggesting necrosis (Fig. 2).

**Fig. 3:**
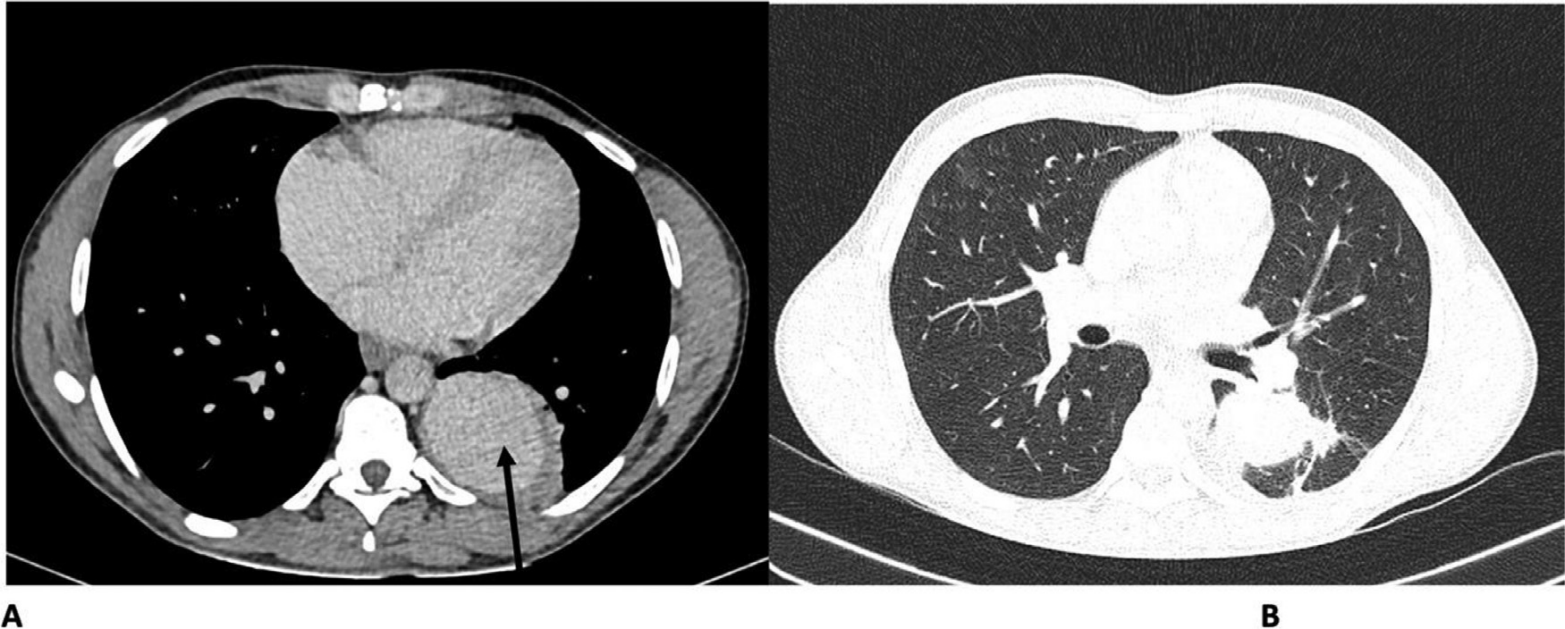
Two months post-treatment CT scan. Contrast-enhanced chest CT scan shows a reduction in the size of the aneurysm (solid arrow in A), measuring 5.8 (TRV) x 5.3 (AP) x 6.8 (CC) cm, with complete resolution of the adjacent left lower lobe cavity. Partial resolution of the left lower lobe consolidation is also seen with reticulations in the superior segment of the left lower lobe suggesting fibrotic changes as seen in B.

The hemoptysis ceased about 2 weeks after starting therapy, and the cough also subsided. Treatment options for RA, including coiling, were discussed with the patient, but conservative management was chosen due to financial constraints. The patient continued the combination of 4 anti-TB antibiotics for a total of 2 months. A follow-up chest CT scan shows a reduction in the size of the aneurysm, at the time measuring 5.8 (TRV) x 5.3 (AP) x 6.8 (CC) cm, with complete resolution of the adjacent left lower lobe cavity. Partial resolution of the left lower lobe consolidation was also seen, with reticulations suggesting fibrotic changes in the superior segment (Fig. 3).

**Fig. 4:**
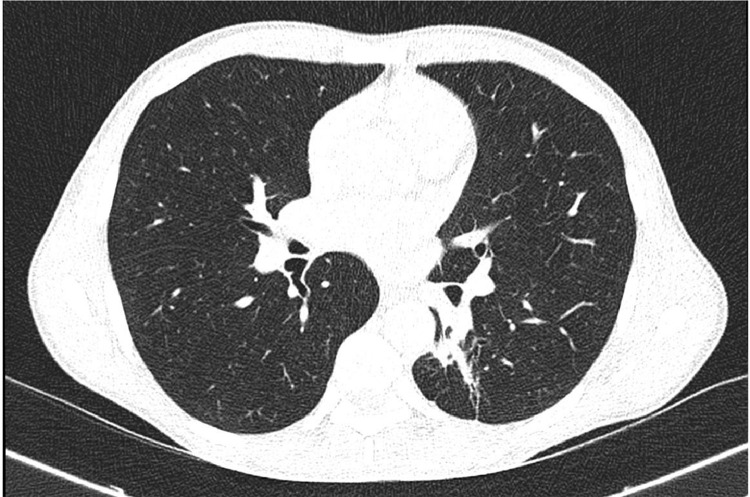
Chest CT done a year and six months later shows complete resolution of the aneurysm and consolidation with mild residual fibrotic changes seen in the superior and posterior segments of the left lower lobe.

According to local TB treatment guidelines, the medication was modified to just Isoniazid and Rifampicin for the next 4 months. The patient started gaining weight, and there were no new symptoms or complaints. After a total of 6 months of anti-TB chemotherapy, the patient was seen and given an appointment for clinical review in 1 year. The patient delayed attending the review due to the lack of complaints. A phone call was made to reiterate the need to attend the review. Another follow-up chest CT scan was performed 18 months after the initial presentation, which showed complete resolution of the aneurysm and consolidation. Mild residual fibrotic changes were seen in the superior and posterior segments of the left lower lobe (Fig. 4).

## Discussion

RAs are pulmonary artery branch pseudo-aneurysms weakening the wall of the artery [4,5] and was first reported in 1868 by Rasmussen et al. [7]. These aneurysms can cause hemoptysis, sometimes severe, and significantly impact morbidity and mortality. It is a relatively uncommon complication of pulmonary TB, and no cases have been published in Ghana according to our literature search.

The most frequently used method for evaluating the cause of hemoptysis is chest radiography and chest CT scan, preferably contrast enhanced scan or CT angiography [4,[8], [9], [10]]. Our patient had chest radiography which was suggestive of pulmonary TB but the RA was diagnosed on subsequent CT, thus underscoring the need for additional imaging in patients with diagnosed or suspected pulmonary TB presenting with hemoptysis.

Typically, there is a long period of time between initial exposure to Mycobacterium tuberculosis and the development of a RA [10], which suggests that our patient experienced a significant delay in diagnosis.

Currently, pulmonary TB is treated in Ghana with a 6-month regimen of antibiotics, including 2 months of Isoniazid, Rifampicin, Pyrazinamide, and Ethambutol, followed by 4 months of Isoniazid and Rifampicin [11]. Our patient completed this treatment regimen.

Treatment options for symptomatic RAs include surgery, such as lobectomy or pneumonectomy [12], or endovascular embolization. In this case, a conservative approach was chosen due to financial constraints and the patient's stable hemodynamic status. After undergoing anti-TB chemotherapy, all symptoms resolved. Furthermore, the area of consolidation, the cavity, and the RA all spontaneously resolved with minor residual architectural changes. Some cases of spontaneous resolution of RAs or mycotic aneurysms have been reported [13].

Therefore, we have demonstrated with this case that conservative management of TB-related RAs in hemodynamically stable patients is a viable option in low-resource settings.

## Conclusion

TB is an endemic disease in Ghana. Hemoptysis in patients diagnosed with pulmonary TB may be due to RA, even though it is uncommon, and should thus be considered in all TB patients presenting with hemoptysis. Contrast enhanced chest CT is a valuable tool in diagnosis. Conservative management of RA in a low resource setting is a viable option that ought to be considered strongly.

## Patient consent

Written informed consent was obtained from the patient for this publication and use of accompanying radiological images.
